# Primary reverse total shoulder arthroplasty in patients aged ≤65 years: a systematic review and meta-analysis

**DOI:** 10.1016/j.xrrt.2026.100722

**Published:** 2026-03-19

**Authors:** Dimitrios Stamiris, Stavros Stamiris, Evangelos M. Solovos, Ioannis Sarris, Georgios Arealis

**Affiliations:** a East Kent Hospitals University Foundation Trust, Kent, UK; b 2^nd^ Orthopaedic Department, 424 General Military Hospital, Thessaloniki, Greece; c 3^rd^Academic Orthopaedic Department, Papageorgiou General Hospital, Aristotle University Medical School, Thessaloniki, Greece

**Keywords:** Primary rTSA, Young adults, Outcomes, Complications, Revision, <65

## Abstract

**Background:**

Reverse total shoulder arthroplasty (rTSA), once reserved for elderly low-demand patients, is increasingly used in younger adults despite concerns regarding complication rates and longevity. This systematic review and meta-analysis evaluate contemporary evidence on primary rTSA in patients aged ≤65 years.

**Methods:**

Following Preferred Reporting Items for Systematic reviews and Meta-Analyses guidelines, PubMed, Scopus, and CENTRAL databases were searched to October 2025. Studies reporting outcomes of primary rTSA in patients ≤65 years with ≥2 years of follow-up were included. Data on indications, functional outcomes, range of motion (ROM), patient-reported outcome measures (PROMs), complications, revisions, and implant survival were recorded and synthesized. Subgroup analyses based on patients' age and comparisons with older rTSA cohorts were also performed.

**Results:**

Thirteen studies met our inclusion criteria. Cuff tear arthropathy (27.29%), massive cuff tears (23.39%), and glenohumeral osteoarthritis (22.34%) accounted for most indications. rTSA resulted in significant improvements in ROM and all major PROMs. The overall complication rate was 14.23%, and the revision rate was 2.92%, with instability and infection responsible for 64.2% of revisions. Mid- to long-term implant survival exceeded 85%. When compared with older patients, younger adults demonstrated equivalent ROM outcomes. Concerning PROMs younger patients demonstrated inferior American Shoulder and Elbow Surgeons (mean difference [MD] = −6.43, *P* = .01), visual analog scale (MD = 0.93, *P* < .01), Constant score (MD = −4.98, *P* < .01), University of California Los Angeles (MD = −2.07, *P* < .01), and Shoulder Pain and Disability Index (MD = 13.56, *P* < .01), whereas Simple Shoulder Test (MD = −0.7, *P* = .19) did not differ between the 2 groups. Moreover, younger patients presented a higher revision risk (risk ratio [RR] = 2.21, *P* < .01).

**Conclusion:**

Primary rTSA provides substantial and predictable improvements in pain and shoulder function among young adults, with acceptable early- to mid-term durability. Nevertheless, younger age remains associated with higher revision risk and modestly lower subjective outcomes, likely reflecting greater functional demands and post-operative expectations. These findings highlight the importance of nuanced surgical decision-making and patient counseling as rTSA use continues to expand in this demographic.

Originally introduced as a salvage procedure for the elderly, low-demand patients, reverse total shoulder arthroplasty (rTSA) has demonstrated favorable early results in treating cuff tear arthropathy (CTA), fracture sequelae, and failed conventional arthroplasties.^9^ Over time, rTSA has become a well-established surgical solution for various complex shoulder conditions, particularly in patients with irreparable rotator cuff tears and CTA, where it reliably restores shoulder function and alleviates pain.^13^

The use of rTSA has expanded markedly over the past 2 decades, driven by broadening indications and an aging patient demographic. National registry data and projection models have documented an exponential rise in rTSA utilization, even among younger patients.^1^ This trend reflects growing surgeon confidence in the reverse prosthesis and its success in various scenarios, but it also raises important questions regarding outcomes in different patient subgroups. In particular, the application of rTSA in younger patients remains somewhat controversial.^20^ In younger patients (commonly defined in the literature as those aged <60 or < 65 years), special considerations apply. These individuals tend to have higher functional demands, longer life expectancy and therefore longer exposure time to the prosthesis, and perhaps different failure modes compared to older patients.^20^ In addition, younger patients typically demonstrate higher activity levels, which may subject rTSA implants to greater cumulative mechanical stress compared with older patients.^20^

There is no uniform definition of a “younger patient” in the rTSA literature, with reported age thresholds ranging from 60 to 70 years.^20^ In the context of rTSA, an age threshold of <65 years is widely adopted in the literature to define “younger” patients because multiple clinical series and reviews have demonstrated that patients up to this age exhibit distinct functional outcomes and complication profiles compared with older cohorts, thereby justifying a primary analysis using this cutoff.^11^ In addition, emerging registry and cohort data indicate that patients aged <60 years present an even higher risk of early revision and inferior clinical outcomes relative to older age groups, supporting an additional, more stringent subgroup analysis at <60 years to elucidate age-dependent differences in complications/revision rates and post-operative function.^29^

A number of systematic reviews have previously addressed rTSA outcomes in younger patients.^6^^,^^11^^,^^15^^,^^36^ Despite these prior efforts, several important gaps remain, justifying the need for a fresh systematic review and meta-analysis. First, the existing reviews often combine primary and revision rTSA cases,^6^^,^^11^^,^^36^ thereby diluting the specific outcomes of primary rTSA in young adults. Second, with the rapid increase in rTSA utilization additional series and longer follow-up (FU) data have emerged, which may allow more precise estimates of outcomes, complications, revisions, and implant survival. Third, important uncertainties remain regarding how patient age influences rTSA results.

The present study therefore aims to systematically investigate the best available evidence concerning primary rTSA in young adults, with the purpose of (1) synthesizing functional outcomes and range of motion gains; (2) quantifying indication for rTSA, complications, reoperations, and revision rates; (3) estimating implant survival where reported; and (4) exploring the published literature for possible differences in rTSA outcomes between younger and older patients, thereby guiding surgical decision-making and patient counseling in this expanding clinical subgroup.

## Materials and methods

### Guidelines followed

This study was conducted following the guidelines of the Preferred Reporting Items for a Systematic Review and Meta-analysis statement.^23^ A completed Preferred Reporting Items for Systematic reviews and Meta-Analyses checklist has been submitted as Supplementary Fig. 1.

### Study selection

The following Population, Intervention, Comparison, Outcome elements were applied for inclusion in this meta-analysis: (i) Population: patients younger than 65 years old, (ii) Intervention: rTSA (any implant design, approach, fixation technique), (iii) Comparative: no comparators in single-arm (pre-operative vs. post-operative outcomes), or parallel cohorts such rTSA in older patients (iv) Outcome: patient-reported outcome measures (PROMs), post-operative range of motion (ROM), complications, and risk for revision.

We enrolled studies that met the following inclusion criteria: (i) were conducted in patients subjected to rTSA; (ii) provided extractable data for young adults (65 years old or younger) treated with primary rTSA; and (iii) had at least 2 years FU for all the included patients. Case-control, case series, cross-sectional, or cohort studies were equally considered for eligibility. A minimum FU period of at least two years was deemed necessary for the evaluation of outcomes after rTSA in patients younger than 65 years, as several clinically relevant complications—such as scapular notching, component loosening, acromial and scapular spine fractures, and baseplate-related mechanical failures—tend to develop or progress over time and may not become apparent within the first post-operative year.^3^^,^^19^

Exclusion criteria were: (i) studies not reporting extractable data on patients <65 years old; (ii) studies reporting on patients with revision rTSA or utilization of custom-made implants; (iii) studies with less than 2 years FU for all the included patients; (iv) Studies not published in English; (v) biomechanical studies; and (vi) animal studies.

### Search strategy

A comprehensive search of PubMed, Scopus, and the Cochrane Central Register of Controlled Trials was performed to identify eligible studies. Our search strategy covered the period from conception until October 17, 2025. The PubMed search string used in our study has been submitted as Supplementary Table 1.

The main search was performed independently by 3 investigators (DS, SS, ES). Any disagreement was resolved by discussion with a third investigator not involved in the initial process (GA). Furthermore, a manual search was conducted throughout reference lists of the included studies.

### Data extraction

The following data were extracted and recorded: (i) first author; (ii) year of publication; (iii) country in which the study was conducted; (iv) study design; (v) number of participants in primary rTSA group, with their demographics; (vi) indication for the primary rTSA; (vii) pre-operative and post-operative ROM and PROMs; (viii) complications, reoperations, and revisions; (ix) 5- and 10-year implant survival rates when available; and (x) additional data concerning comparison of primary study group with rTSA in older patients, hemiarthroplasty, or aTSA.

### Data imputation

In cases of missing data, a stepwise approach was implemented. Initially, we attempted to contact the authors via e-mail to acquire the necessary information. If this was unsuccessful, appropriate statistical methods were employed to estimate the missing values and ensure a comprehensive analysis.^17^ Finally, if missing data could not be addressed through these methods, the studies were excluded from the analysis of the outcome. In particular, in 2 studies standard deviations were derived from means and 95% confidence intervals (CI) or mean difference (MD) and *P* value between groups.^17^

### Risk of bias and study quality assessment

Quality assessment of the included studies was performed independently by the same 3 authors, using the Methodological Index for Nonrandomized Studies (MINORS).^34^ MINORS is an instrument designed to evaluate the methodological quality of nonrandomized surgical studies, whether comparative or noncomparative. It focuses on different aspects of study design, conduct, and reporting. The MINORS tool consists of 12 domains. Each domain can receive a score of 0-2 points (no information reported, not adequate information reported, adequate information reported). The first 8 domains apply to noncomparative studies, with a maximum score of 16, whereas for controlled studies, 4 additional domains are included, yielding a maximum score of 24. Higher MINORS points correspond to a higher study quality with lower risk of bias. For noncomparative studies, quality was considered poor at a score ≤8, moderate at 9-14, and good at 15-16, whereas for comparative studies the corresponding scores were ≤14, 15-22, and ≥ 23, respectively.

### Statistical analysis

A random-effects model was used for data synthesis (Mantel-Haenszel model) in cases of moderate to high heterogeneity or fixed effects model in cases of low heterogeneity. Heterogeneity was tested by the Cochrane chi-square test. The degree of heterogeneity was evaluated with the I^2^ statistics; heterogeneity was defined as low (I^2^<30%), moderate (I^2^ = 30%-60%), or high (I^2^> 60%). Associations were reported as odds ratios with 95% CI for qualitative variables and as MDs with 95% CI for quantitative measurements. When measurement methods were inconsistent (internal rotation), standardized MD (SMD) with 95% CI was used to adjust for differences in measurement scales. Subgroup analyses were undertaken to explore the results of primary rTSA outcomes between different age groups (<60, <65). In cases where high heterogeneity was encountered, additional sensitivity analyses were made to locate outliers, defined as studies that had lower methodological quality or those in risk for adding substantial heterogeneity. In detail, sensitivity analysis was implemented by removing the studies that included patients treated for fractures (a well-known factor for poorer post-operative outcomes).^18^^,^^28^ Moreover, in analyses where >50% of patients were provided by a single study, additional sensitivity analysis was made by excluding this study to assess the robustness of our results. Finally, an additional sensitivity analysis was made by removing the studies with imputed data (Supplementary Fig. 2).

A minimum of 2 studies reporting the outcomes of interest were required to perform a subgroup or sensitivity analysis. Publication bias was assessed using Funnel plots. All analyses were performed using Review Manager 5.4 software (Cochrane Library).

## Results

### Literature search and selection process

**Figure 1:**
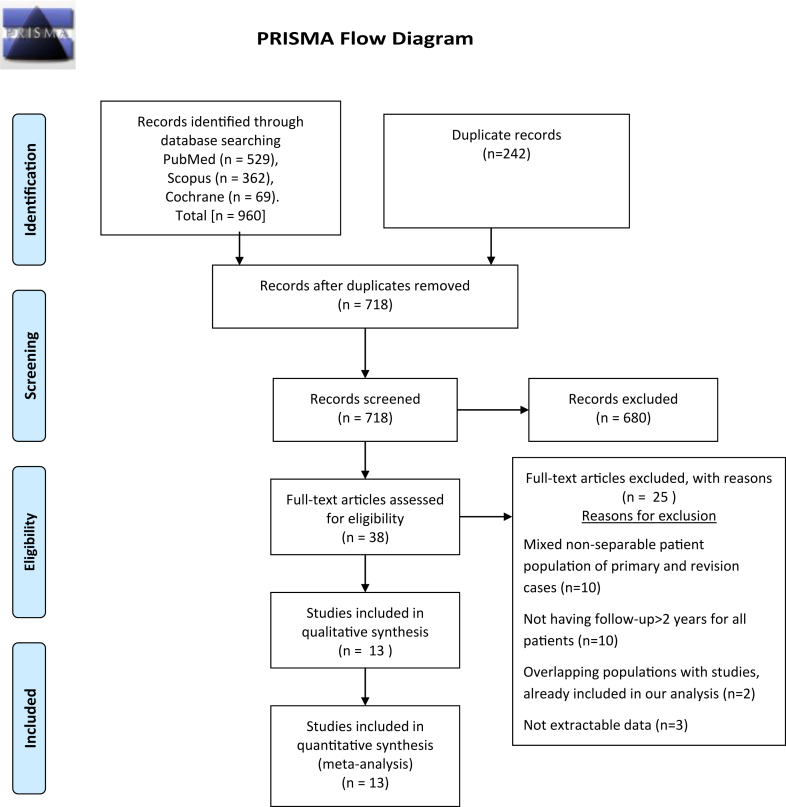
PRISMA flow diagram illustrating the study selection process. *PRISMA*, Preferred Reporting Items for Systematic reviews and Meta-Analyses.

Initial search yielded 960 potentially relevant studies. Following removal of duplicates (n = 242), 718 studies were screened based on title and abstract. Full-text assessment was conducted in 38 studies, 25 of which were excluded for the following reasons: (i) 10 studies reported on mixed nonseparable patient population of primary and revision cases; (ii) 10 studies had FU less than 2 years,; (iii) 2 studies had overlapping population with other studies already included in our analysis; and (iv) 3 studies had nonextractable data. A flowchart diagram of the search strategy is shown in Fig. 1A, complete list of excluded studies with reasons have been submitted as Supplementary Table 2.

### Study characteristics

Thirteen studies were eventually eligible for qualitative and quantitative analysis, based on our pre-established criteria. Eleven of these studies were conducted in USA,^4^^,^^8^^,^^10^^,^^12^^,^^21^^,^^24^^,^^26^^,^^27^^,^^29^^,^^30^^,^^32^ 1 in France,^7^ and 1 study was conducted in Switzerland.^14^ Seven studies evaluated patients younger than 60 years of age,^4^^,^^7^^,^^10^^,^^14^^,^^26^^,^^27^^,^^29^ whereas 6 studies reported on patients younger than 65.^8^^,^^12^^,^^21^^,^^24^^,^^30^^,^^32^ Overall, all the included studies comprised a total population of 5,333 patients ≤65 years old, treated with a primary rTSA. The mean age of the included patients was 55.1 years. All the included studies had minimum FU of two years for all the included patients (as per our inclusion criteria). The mean FU ranged from 2.7 to 11.7 years. A summary of the descriptive characteristics of the included studies is presented in Table I.

**Table I:** Descriptive characteristics of the included studies.

ID	1st author, yr	Study design	Country	Young adults age group	Indication for primary rTSA	N	Mean age	M/F	Mean FU
1	Panel, 2025	Retrospective cohort	USA	<60	NR	4,634	55.8	2,029/2,605	
2	Barry, 2025	Retrospective, case-control	USA	<60	RCT: 30, GHOA: 6, fracture seq: 5, instability: 5, tumor: 2, AVN: 1, inflam: 1	50	56.9	20/30	4.9
3	Berhouet, 2024	Retrospective case series	France	<60	RCT: 30, fracture seq: 37, tumor: 18, CTA: 17, inflam: 15, instability: 11, GHOA: 8	136	51.6	53/83	6.5
4	Deliso, 2023	Retrospective cohort	USA	<65	CTA: 12, fracture seq: 7	19	57.4	9/10	2.7
5	Neel, 2022	Prospective, case-control	USA	<60	GHOA: 78 (31 no cuff deficiency - 48 deficient cuff), CTA: 39, post-traumatic (no-fracture): 17, inflamm: 20	154	55	69/85	4
6	Shah, 2021	Retrospective cohort	USA	<65	GHOA (all had cuff deficiency or notable glenoid deformity), CTA	31			
7	Monir, 2020	Retrospective case series	USA	<65	CTA: 15, RCT: 12, GHOA: 10 (all had cuff deficiency), fracture seq; 7, inflam: 6, tumor: 1, AVN: 1	52	58	17/35	6.3
8	Brewley, 2020	Retrospective cohort	USA	<60	CTA: 15, GHOA: 17 (4/17 had cuff tear), RCT: 15, instability: 3, inflam: 8∗	62			4.16
9	Matthews, 2019	Retrospective, case-control	USA	<65	CTA: 23, GHOA: 11 (all had cuff deficiency), fracture seq: 4, RCT: 2, inflamm: 3	43	60.4	17/26	4
10	Ernstbrunner, 2017	Retrospective case series	Switzerland	<60	RCT: 23	20†	57	11/9	11.7
11	Samuelsen, 2017	Retrospective case series	USA	<65	CTA: 51, GHOA: 15 (severe arthritis), AVN: 1	67	60	27/40	3
12	Otto, 2017	Retrospective, case-control	USA	<60	CTA: 10, RCT: 11, fracture seq: 5, GHOA: 4, inflam: 2	32‡	48.9	19/13	4.98
13	Black, 2014	Retrospective, case-control	USA	<65	RCT	33	59.3	9/24	4.55

*M*, male; *F*, female; *rTSA*, reverse total shoulder arthroplasty; *N*, number of patients; *FU*, follow-up (mean) in yr; *NR*, not reporting; *RCT*, rotator cuff tear; *GHOA*, glenohumeral osteoarthritis; *seq*, sequelae; *CTA*, cuff tear arthropathy; *AVN*, avascular necrosis; *inflam*, inflammatory arthropathy.

∗ reporting indications for rTSA for the 58 patients who did not undergo revision.

† 20 patients/23 shoulders.

‡ Only data from primary subgroup used.

### Risk of bias and publication bias assessment

Risk of bias assessment was conducted using the MINORS criteria. Overall, all the included studies were deemed to be of moderate quality. In detail, the 6 noncomparative studies achieved scores between 9 and 12 points, whereas the remaining 7 comparative studies achieved scores between 15 and 19 points. The quality assessment of the included studies using the MINORS score is depicted in Supplementary Table 3. Concerning publication bias assessment, visual inspection of the funnel plots did not show an increased risk for publication bias.

### Indications for primary reverse total shoulder arthroplasty in young adults

Extractable data regarding indication for the primary rTSA were available from 11 of the included studies.^4^^,^^7^^,^^8^^,^^10^^,^^12^^,^^14^^,^^21^^,^^24^^,^^26^^,^^27^^,^^30^ The most common indication for primary rTSA in young adults was CTA (27.29%), followed by massive rotator cuff tear (MRCT) (23.39%) and primary glenohumeral osteoarthritis (22.34%). Less frequent indications included fracture sequelae (9.75%), inflammatory arthropathy (8.25%), tumor (3.15%), postinstability arthropathy (2.85%), post-traumatic arthritis (2.53%), and avascular necrosis (0.45%).

Based on data from 5 studies,^10^^,^^21^^,^^24^^,^^26^^,^^30^ which explicitly report the content of the glenohumeral osteoarthritis group among the patients treated with rTSA, 67.18% of these patients had either cuff deficiency or severe arthritis with substantial glenoid and/or humeral deformity.

### Outcomes of primary reverse total shoulder arthroplasty in young adults

#### ROM and PROMs

**Figure 2:**
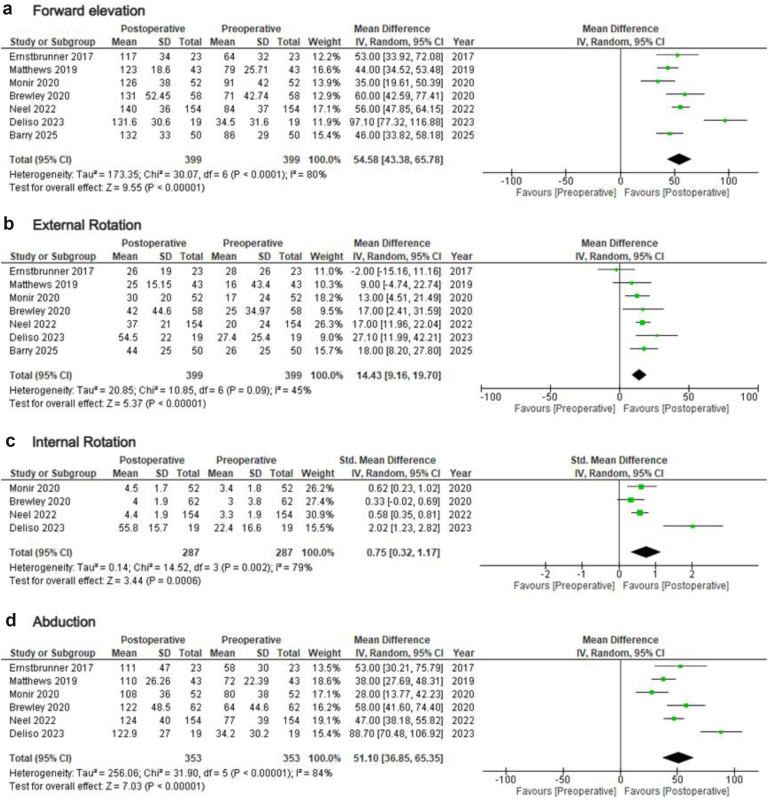
Forest plots demonstrating post-operative improvements in range of motion (ROM) after primary rTSA in young adults. (**a**) Forward elevation, (**b**) external rotation, (**c**) internal rotation, and (**d**) abduction. *rTSA*, reverse total shoulder arthroplasty; *SD*, standard deviation; *CI*, confidence interval; *df*, degrees of freedom.

Data regarding ROM improvement following rTSA in patients younger than 65 were available from 11 studies^4^^,^^7^^,^^8^^,^^10^^,^^12^^,^^14^^,^^21^^,^^24^^,^^26^^,^^27^^,^^30^ (Table II). Overall, rTSA significantly improved patients forward elevation (FE) (MD = 54.58; 95% CI = 43.38-65.78; *P* < .01; I2: 80%), external rotation (ER) (MD = 14.43; 95% CI = 9.16-19.70; *P* < .01; I2: 45%), internal rotation (IR) (SMD = 0.75; 95% CI = 0.32-1.17; *P* = .02; I2: 79%), and abduction (ABD) (MD = 51.1; 95% CI = 36.85-65.35; *P* < .01; I2: 84%) (Fig. 2). Sensitivity analyses, by removing the studies that evaluated patients with fractures, produced same results, with improved heterogeneity (FE: MD = 56.23; 95% CI = 49.35-63.12; *P* < .01; I2: 0%/IR: SMD = 0.51; 95% CI = 0.31-0.70; *P* < .01; I2: 24%/ABD: MD = 49.84; 95% CI = 42.48-57.19; *P* < .01; I2: 0%) (Supplementary Fig. 3). Subgroup analyses for the same outcomes in the studies evaluating patients younger than 60 years also showed similar results (FE: MD = 53.75; 95% CI = 47.76-59.75; *P* < .01; I2: 0%/ER: MD = 13.55; 95% CI = 5.74-21.36; *P* < .01; I2: 60%/IR: SMD = 0.51; 95% CI = 0.31-0.70; *P* < .01; I2: 24%/ABD: MD = 49.84; 95% CI = 42.48-57.19; *P* < .01; I2: 0%) (Supplementary Fig. 3).

**Table II:** Outcomes of primary rTSA in young adults.

Study	Complications	Revision	5-yr ISR	10-yr ISR	ROM	ASES	VAS	Constant	SST	UCLA	SPADI	SSV
					PRE	PRE	PRE	PRE	PRE	PRE	PRE	PRE
					POST	POST	POST	POST	POST	POST	POST	POST
Panel		117										
Barry	12%	6	85.8%	85.8%	FE: 86 ± 29, ER: 26 ± 25, IR: S1							
					FE: 132 ± 33, ER: 44 ± 25, IR: L5	78.9 ± 19.8	2.3 ± 3		8.0 ± 3.3			
Berhouet∗	27%				FE: 79.7, ER: 13.8, IR: 4.1			32.2				
					FE: 128.3, ER: 16.9, IR: 4.8			57.7				
Deliso		2			FE: 34.5 ± 31.6, ER: 27.4 ± 25.4, IR: 22.4 ± 16.6, ABD: 34.2 ± 30.2							
					FE: 131.6 ± 30.6, ER: 54.5 ± 22.2, IR: 55.8 ± 15.7, ABD: 122.9 ± 27							
Neel∗	4.5%	7			FE: 84 ± 37, ER: 20 ± 24, IR: 3.3 ± 1.9, ABD: 77 ± 39	32 ± 15	6.5 ± 2.1	32 ± 13	2.8 ± 2.6	12 ± 4.2	91 ± 21	
					FE: 140 ± 36, ER: 37 ± 21, IR: 4.4 ± 1.9, ABD: 124 ± 40	72 ± 24	2.3 ± 2.6	64 ± 20	8.5 ± 3.5	28 ± 7.0	38 ± 34	
Shah						72.7 ± 22						
Monir∗	7.7%	3			FE: 91 ± 42, ER: 17 ± 24, IR: 3.4 ± 1.8, ABD: 80 ± 38	33.5 ± 14.5	6.3 ± 2.3	33.6 ± 12.7	3 ± 2.2	13.0 ± 4.2	87.4 ± 19.0	
					FE: 126 ± 38, ER: 30 ± 20, IR: 4.5 ± 1.7, ABD: 108 ± 36	74.3 ± 21.5	1.7 ± 2.2	62.8 ± 18.7	8.7 ± 3.3	27.5 ± 7.0	37.9 ± 34.8	
Brewley†		4			FE: 71 ± 42.74, ER: 25 ± 34.97, IR: 3 ± 3.8, ABD: 64 ± 44.6	30 ± 15.54						
					FE: 131 ± 52.45, ER: 42 ± 44.6, IR: 4 ± 1.9, ABD: 122 ± 48.5	62 ± 25.5						
Matthews‡	5%	0			FE: 79 ± 25.71, ER: 16 ± 43.4, IR: L4, ABD: 72 ± 22.39	32 ± 15.13		33.3 ± 10.2	2.5 ± 2.8	12.6 ± 3.1	94.5 ± 16.0	
					FE: 123 ± 18.6, ER: 25 ± 15.15, IR: L2, ABD: 110 ± 26.26	71.2 ± 18		69.6 ± 13.6	8.5 ± 2.6	27 ± 2.1	40.5 ± 22.9	
Ernstbrunner	39%	4	96%	92%	FE: 64 ± 32, ER: 28 ± 26, ABD: 58 ± 30			24 ± 9				20 ± 13
					FE: 117 ± 34, ER: 26 ± 19, ABD: 111 ± 47			59 ± 19				71 ± 27
Samuelsen	9%	2	91%		ABD: 57.5, ER: 20.1							
					ABD: 132.4, ER: 39.4	62 ± 16			5.9 ± 3			
Otto	18.7%	4			FE: 64.8, ER: 11.3, IR: GT, ABD: 45.6	28.1			1.3			
					FE: 113.2, ER: 30, IR: L3-L4, ABD: 79	58.6			4.5			
Black	18.2%	2					7 ± 1.6		1.9 ± 1.4			19 ± 14
					FE: 112 ± 47, ER: 35 ± 14	74.0 ± 23.8	2.1 ± 3.3		7.6 ± 2.6			6 ± 26

*rTSA*, reverse total shoulder arthroplasty; *ASES*, American Shoulder and Elbow Surgeons; *VAS*, visual analog scale; *SST*, Simple Shoulder Test; *UCLA*, University of California Los Angeles; *SSV*, Subjective Shoulder Value; *SPADI*, Shoulder Pain and Disability Index; *ISR*, implant survival rate; *PRE*, pre-operative; *POST*, post-operative; *FE*, forward elevation; *ER*, external rotation; *IR*, internal rotation; *ABD*, abduction; *SD*, standard deviation; *CI*, confidence interval; *MD*, mean difference; *GT*, greater trochanter; *ROM*, range of motion.

∗ Internal rotation was measured using a point system.

† Internal rotation was measured using a point system and SD was calculated from mean and 95% CI using the method described in: Higgins JPT, Thomas J, Chandler J, Cumpston M, Li T, Page MJ, Welch VA (editors). Cochrane Handbook for Systematic Reviews of Interventions version 6.5 (updated August 2024). Cochrane, 2024. Available from www.cochrane.org/handbook.

‡ Internal rotation was measured using a point system and SD was calculated using MD, *P* value, and *t*-statistic, as described in: Higgins JPT, Thomas J, Chandler J, Cumpston M, Li T, Page MJ, Welch VA (editors). Cochrane Handbook for Systematic Reviews of Interventions version 6.5 (updated August 2024). Cochrane, 2024. Available from www.cochrane.org/handbook.

**Figure 3:**
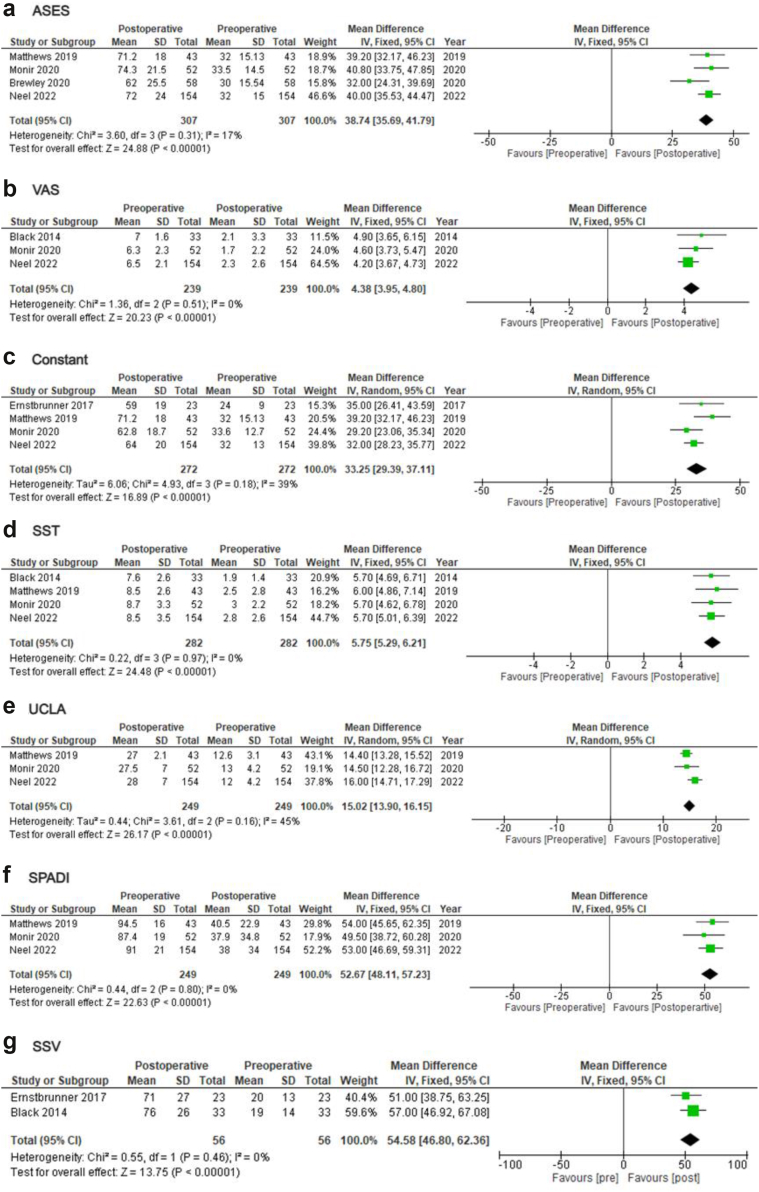
Forest plots displaying pooled changes in PROMs following primary rTSA in young adults. (**a**) ASES, (**b**) VAS pain, (**c**) Constant score, (**d**) SST, (**e**) UCLA, (**f**) SPADI, and (**g**) SSV. *rTSA*, reverse total shoulder arthroplasty; ASES, American Shoulder and Elbow Surgeons; *VAS*, visual analog scale; *SST*, Simple Shoulder Test; *UCLA*, University of California Los Angeles; *SSV*, Subjective Shoulder Value; *SPADI*, Shoulder Pain and Disability Index; *SD*, standard deviation; *df*, degrees of freedom; *CI*, confidence interval; *PROM*, patient-reported outcome measure.

Eleven studies reported on PROMs alterations following rTSA in young adults^4^^,^^7^^,^^8^^,^^10^^,^^12^^,^^14^^,^^21^^,^^24^^,^^26^^,^^27^^,^^30^ (Table II). rTSA led to an improvement in post-operative American Shoulder and Elbow Surgeons (ASES) (MD = 38.74; 95% CI = 35.69-41.79; *P* < .01; I2: 17%), visual analog scale (VAS) (MD = 4.38; 95% CI = 3.95-4.80; *P* < .01; I2: 0%), Constant score (MD = 33.25; 95% CI = 29.39-37.11; *P* < .01; I2: 39%), Simple Shoulder Test (SST) (MD = 5.75; 95% CI = 5.29-6.21; *P* < .01; I2: 0%), University of California Los Angeles (UCLA) (MD = 15.02; 95% CI = 13.90-16.15; *P* < .01; I2: 45%), Shoulder Pain and Disability Index (SPADI) index (MD = 52.67; 95% CI = 48.11-57.23; *P* < .01; I2: 0%), and Shoulder Subjective Value (MD = 54.58; 95% CI = 46.8-62.36; *P* < .01; I2: 0%) (Fig. 3). In the subgroup analyses for the <60 subgroup, results remained unchanged (ASES: MD = 36.64; 95% CI = 28.90-44.38; *P* < .01; I2: 68%/Constant: MD = 32.48; 95% CI = 29.03-35.93; *P* < .01; I2: 0%) (Supplementary Fig. 4).

#### Complications and revision rate

Nine studies reported outcomes regarding post-operative complications (590 patients).^4^^,^^7^^,^^8^^,^^14^^,^^21^^,^^24^^,^^26^^,^^27^^,^^30^ Overall, the complication rate for patients younger than 65 years old treated with a primary rTSA was 14.23%. In the ≤60 subgroup, this rate was slightly higher at 16.7%.^4^^,^^7^^,^^14^^,^^26^^,^^30^ Concerning revision rate, data were available from 11 studies (5,169 patients).^4^^,^^8^^,^^10^^,^^12^^,^^14^^,^^21^^,^^24^^,^^26^^,^^27^^,^^29^^,^^30^ The aggregate rate of revision for young adults (≤65) following rTSA was 2.92%. A similar rate was noted from the studies evaluating patients ≤60 (2.86%).

Instability and infection represented the leading causes for revision, being responsible in total for the 64.2% of the cases (32.1% each). Implant loosening was the third most common reason with a rate of 10.7%, followed by implant dissociation at 7.14%. Less common reasons included periprosthetic fracture, persistent pain, and nerve injury.

Data on implant mid- and long-term survival rate were reported by 3 studies.^4^^,^^14^^,^^30^ In particular, 5-year implant survival ranged from 85.8% to 96%,^4^^,^^14^^,^^30^ and 10-year implant survival was reported to be 85.8%.^4^

### Comparison of outcomes between reverse total shoulder arthroplasty in younger and older adults

Seven studies reported comparative outcomes between younger and older patients treated with primary rTSA.^4^^,^^10^^,^^12^^,^^21^^,^^26^^,^^29^^,^^32^ Three of these studies considered 65 years of age as the limit between younger and older groups,^12^^,^^21^^,^^32^ while the remaining 4 contemplated 60 years Table I. Data concerning the older rTSA groups are depicted in Supplementary Table 4.

**Figure 4:**
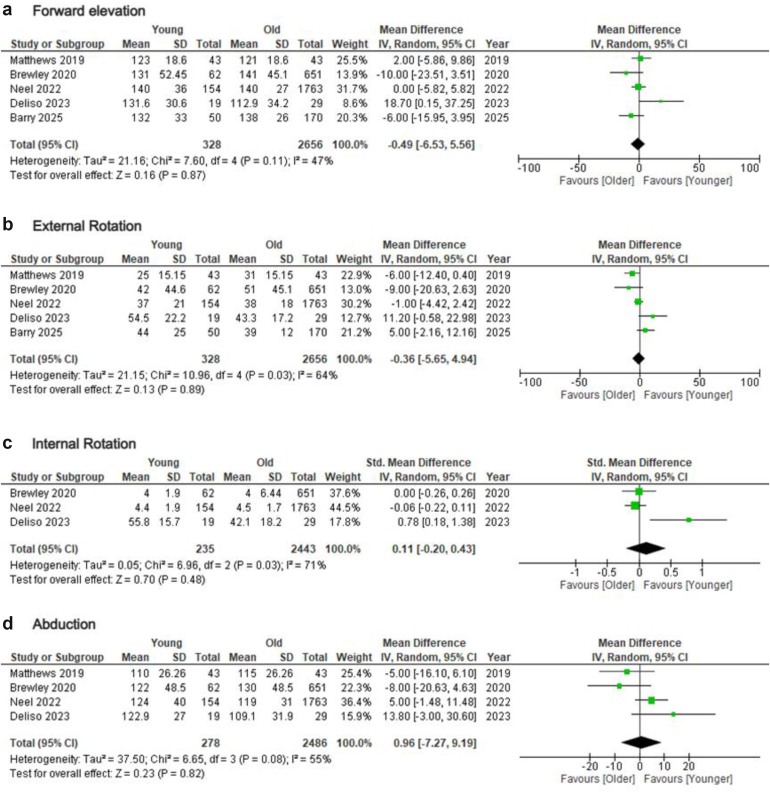
Forest plots comparing post-operative ROM between younger adults and older adults undergoing primary rTSA. (**a**) forward elevation, (**b**) external rotation, (**c**) internal rotation, and (**d**) abduction. *rTSA*, reverse total shoulder arthroplasty; *SD*, standard deviation; *CI*, confidence interval; *df*, degrees of freedom; *ROM*, range of motion.

Both groups of patients (younger and older) demonstrated similar post-operative ROM in all planes. In particular, no difference was found regarding FE (MD = −0.49; 95% CI = −6.53-5.56; *P* = .87; I2: 47%), ER (MD = −0.36; 95% CI = −5.65-4.94; *P* = .89; I2: 64%), IR (SMD = 0.11; 95% CI = −0.20-0.43; *P* = .48; I2: 71%), and ABD (MD = 0.96; 95% CI = −7.27-9.19; *P* = .82; I2: 55%) (Fig. 4). Sensitivity analysis, by excluding studies that evaluated patients with fractures, again revealed no difference between younger and older patients with improved heterogeneity (ER: MD = 2.91; 95% CI = −3.77-9.59; *P* = .39; I2: 40%/IR: SMD = 0.10; 95% CI = −0.18-0.38; *P* = .49; I2: 0%) (Supplementary Fig. 5). Subgroup analysis, by removing the studies that considered patients between 60 and 65 years old in the older group (evaluating only studies comparing patients younger than 65 to older), showed similar results (FE: MD = 8.15; 95% CI = −7.64-23.94; *P* = .31; I2: 62%/ER: MD = 1.86; 95% CI = −14.93-18.65; *P* = .83; I2: 84%/ABD: MD = 3.30; 95% CI = −15.0-21.6; *P* = .72; I2: 70%) (Supplementary Fig. 5).

**Figure 5:**
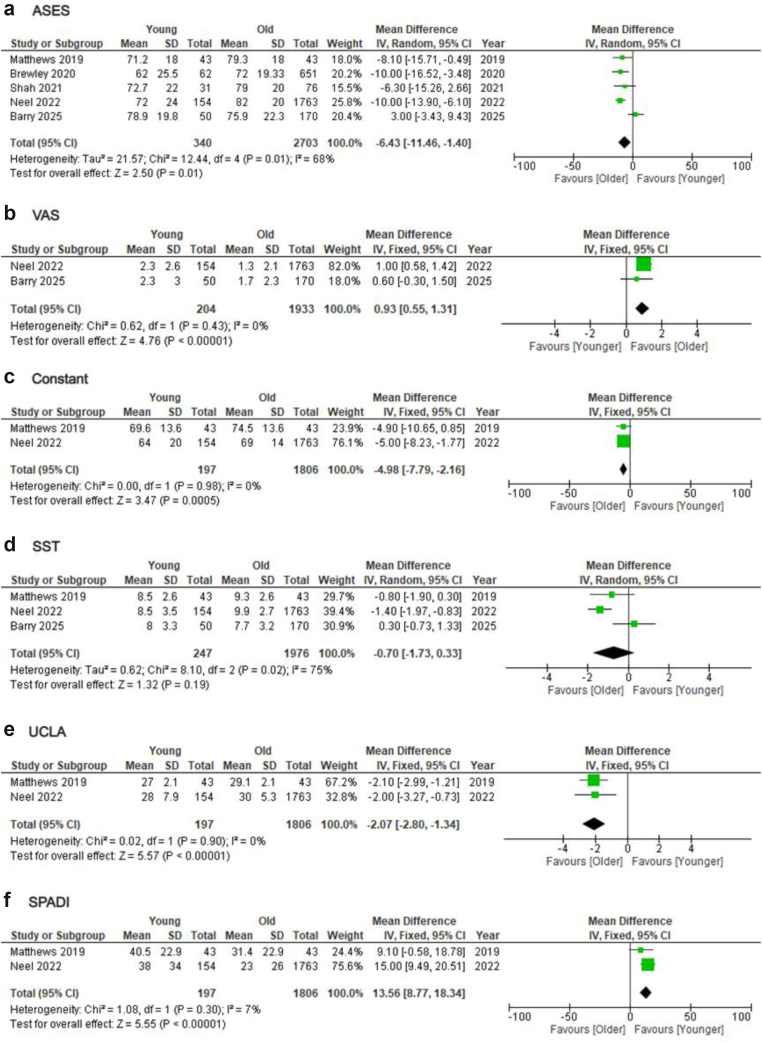
Forest plots comparing post-operative PROMs between younger and older patients undergoing primary rTSA. (**a**) ASES, (**b**) VAS pain, (**c**) Constant score, (**d**) SST, (**e**) UCLA, and (**f**) SPADI. *rTSA*, reverse total shoulder arthroplasty; *ASES*, American Shoulder and Elbow Surgeons; *VAS*, visual analog scale; *SST*, Simple Shoulder Test; *UCLA*, University of California Los Angeles; *SSV*, Subjective Shoulder Value; *SPADI*, Shoulder Pain and Disability Index; *SD*, standard deviation; *CI*, confidence interval; *PROM*, patient-reported outcome measure; *df*, degrees of freedom.

Concerning PROMs, data were available from 5 studies.^4^^,^^10^^,^^12^^,^^21^^,^^26^ Younger group demonstrated worse post-operative ASES (MD = −6.43; 95% CI = −11.46 to −1.40; *P* = .01; I2: 68%), VAS (MD = 0.93; 95% CI = 0.55-1.31; *P* < .01; I2: 0%), Constant score (MD = −4.98; 95% CI = −7.79 to −2.16; *P* < .01; I2: 0%), UCLA (MD = −2.07; 95% CI = −2.8 to −1.34; *P* < .01; I2: 0%), and SPADI index (MD = 13.56; 95% CI = 8.77-18.34; *P* < .01; I2: 7%), whereas SST (MD = −0.7; 95% CI = −1.73-0.33; *P* = .19; I2: 75%) did not differ between the 2 groups (Fig. 5). Sensitivity analysis, by removing studies that included patients with a fracture, again found significantly worse post-operative ASES score for the younger group, with improved heterogeneity (MD = −9.55; 95% CI = −12.68 to −6.41; *P* < .01; I2: 0%) (Supplementary Fig. 6). Subsequent subgroup analysis, comparing patients younger than 65 to older, revealed identical results (ASES: MD = −7.35; 95% CI = −13.14 to −1.55; *P* = .01; I2: 0%) (Supplementary Fig. 6).

**Figure 6:**
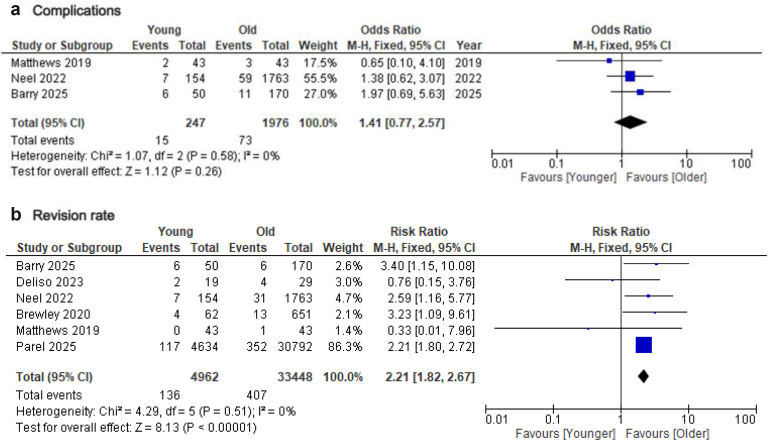
Forest plots comparing post-operative adverse outcomes between younger and older adults following primary reverse total shoulder arthroplasty. (**a**) complications and (**b**) revision rate. *M-H*, Mantel-Haenszel; *CI*, confidence interval; *df*, degrees of freedom.

With respect to complications and revision rate, data were available from 6 studies.^4^^,^^10^^,^^12^^,^^21^^,^^26^^,^^29^ No difference was found regarding complications rate between younger and older patients (odds ratio = 1.41; 95% CI = 0.77-2.57; *P* = .26; I2: 0%), whereas the risk for revision was significantly higher in the younger group (RR= 2.21; 95% CI = 1.82-2.67; *P* < .01; I2: 0%) (Fig. 6). Subsequent sensitivity analysis by removing the study by Parel et al (which distributed >50% of patients regarding revision rate), found similar results (RR = 2.21; 95% CI = 1.34-3.64; *P* < .01; I2:7%) (Supplementary Fig. 7).

## Discussion

This systematic review and meta-analysis synthesize the best available evidence on primary rTSA in young adults (≤65 years old). As implementation of rTSA in younger individuals continues to gain popularity, understanding the true performance and the contemporary risks of primary implants in this active and demanding population is critical for evidence-based clinical decision-making.^20^ Our analysis of 13 studies encompassing 5,333 patients provides several important insights regarding indications, functional outcomes, complications, and implant survival in this increasingly relevant patient subgroup.

The results of this review confirm that primary rTSA yields substantial and clinically meaningful improvements in function and pain relief among younger adults. Significant gains were consistently observed in all major planes of motion—FE, ABD, ER, and IR—demonstrating that rTSA reliably restores functional arcs necessary for activities of daily living. Improvements across multiple PROMs similarly demonstrated significant enhancements, with consistent improvements in ASES, Constant score, UCLA score, VAS, SPADI, and SST. It should be noted that across all evaluated outcome measures, improvement exceeded the Minimum Clinically Importance Difference.^33^^,^^35^ These findings underscore that younger patients not only experience objective functional restoration but also report marked subjective benefit.

Our findings are consistent with, yet extend beyond, previous systematic reviews that have investigated primary rTSA outcomes in younger populations.^6^^,^^11^^,^^15^^,^^36^ All the aforementioned studies have reported satisfactory functional outcomes after rTSA in younger patients, though these earlier reviews (with the exception of the study by Goldenberg et al) often combined primary and revision cases, thereby obscuring the true results of primary procedures. By restricting our analysis to primary rTSA cases and incorporating contemporary series with longer FU, the present review provides more accurate and granular estimates of outcomes in young adults.

In terms of complication and revision rates, our pooled complication (14.23%) and revision rates (2.92%) are lower than that reported by Goldenberg et al,^15^ possibly reflecting the inclusion of more recent implant designs, improved surgical technique, and better patient selection in the newer studies covered by our analysis.

Despite demonstrating comparable ROM outcomes between younger and older patients, our analysis identified differences in patient-reported outcomes. Younger adults exhibited slightly inferior PROMs compared with older adults, despite equivalent objective motion and complication rates. This discrepancy aligns with observations by Muh et al^25^ and Henn et al,^16^ who hypothesized that younger, more active individuals have higher baseline functional demands and expectations and may perceive smaller improvements as less satisfactory despite comparable objective function. This “expectation gap” may partly explain why ASES, VAS, and Constant scores, although improved, remain modestly lower in younger adults than in their older counterparts. The latter also emphasizes the need for in-depth pre-operative assessment of patient expectations and counseling to align patient goals with surgical realities.

Instability and infection remain the leading causes of revision in younger patients, consistent with registry data.^22^^,^^25^ The mid- and long-term survivorship figures (5- and 10-year implant survival rates) are also in line with recently published registry data^22^^,^^37^ reporting close to 93% 5-year implant survival and <91% 10 years, suggesting acceptable durability given the functional demands of this population.

These findings provide reassuring evidence that primary rTSA can deliver excellent pain relief, restoration of motion, and durable implant survival in younger patients, challenging the traditional notion that rTSA should be reserved for the elderly, low-demand population. The similar ROM outcomes between younger and older cohorts suggest that prosthesis biomechanics and soft-tissue tensioning principles translate effectively across age groups.

However, the higher revision rate and slightly lower PROMs among younger adults, compared to older (>65 years old) patients, emphasize the importance of careful patient selection and counseling. Surgeons should discuss realistic expectations regarding implant longevity (including 5- and 10-year survival rate) and the potential need for revision surgery in the long term. Given that younger patients are likely to outlive their prosthesis, biological bone preservation and implant modularity should be prioritized when planning surgery, and alternative treatments (eg, tendon transfers, biologic augmentation, or anatomic arthroplasty) may remain preferable options in select cases.^2^^,^^5^^,^^31^

This meta-analysis presents several limitations inherent to its study design and to the available evidence. Most of the included studies were retrospective case series or cohort studies with moderate methodological quality (MINORS scores between 9 and 12 for noncomparative studies and 15 and 19 for comparative), which limits the strength of causal inference. Heterogeneity across studies was considerable for some ROM outcomes, likely reflecting differences in indication for the initial surgery (eg, fracture vs. elective), implant design, surgical approach, rehabilitation protocols, and patient activity levels. Using sensitivity analyses, we identified fracture sequelae as a major source of heterogeneity (removing studies evaluating patients with fracture sequelae as an indication for rTSA reduced the observed heterogeneity of the results), but other factors may also contribute. Additional sensitivity analyses evaluating the outcomes of rTSA in this younger subgroup of patients based on surgical indication, implant type, surgical technique and method of fixation, or post-operative course (including rehabilitation and return to work) could not be performed due to insufficient data reported in the included studies. Furthermore, FU duration varied (2.7-11.7 years), and only 3 studies provided mid- to long-term survival data, restricting precise assessment of prosthesis performance in the long term. Yet, the major strength of our study is the systematic and comprehensive search of the available literature to identify studies exploring the results, complications, and revision rates of primary rTSA in younger adults.

## Conclusion

Primary rTSA in younger adults yields substantial functional gains, pain relief, and acceptable mid-term implant survival, comparable to older patients. However, younger age remains associated with higher revision risk and slightly lower subjective satisfaction, possibly reflecting the unique biomechanical and expectation-related challenges in this group. Surgeons should individualize treatment, balancing the predictable benefits of rTSA against the potential need for revision in the patient's lifetime. Continued surveillance and further high quality studies with long-term outcome reporting are warranted to optimize care for this growing population of younger rTSA recipients.

## Availability of data

Data can be provided upon request.

## Disclaimers:

Funding: No funding was received for conducting this study. The publication of this article in OA mode was financially supported by HEAL-Link.

Conflicts of interest: The authors, their immediate families, and any research foundations with which they are affiliated have not received any financial payments or other benefits from any commercial entity related to the subject of this article.
