# Supplementary material for: Primary reverse total shoulder arthroplasty in patients aged ≤65 years: a systematic review and meta-analysis
Source: JSES Rev Rep Tech. 2026 Mar 19;6(3):100722. doi: 10.1016/j.xrrt.2026.100722 (PMC13092040; doi:10.1016/j.xrrt.2026.100722)
Supplement: Supplementary Table 1 [file mmc1.docx]

| **Supplementary Table 1.** |
| --- |
| **PubMed search string** |
| ("reverse shoulder arthroplasty"[tiab] OR "reverse total shoulder arthroplasty"[tiab] OR "RTSA"[tiab] OR "reverse shoulder replacement"[tiab] OR "reverse total shoulder replacement"[tiab] OR "reverse shoulder prosthesis"[tiab] OR "reverse shoulder arthroplasty"[MeSH])AND(young[tiab] OR younger[tiab] OR "young adult"[MeSH] OR "early-onset"[tiab] OR "under 65"[tiab] OR "middle-aged"[tiab] OR "age < 65"[tiab] OR "≤65"[tiab])AND(surviv*[tiab] OR "implant survival"[tiab] OR "prosthesis survival"[tiab] OR revision[tiab] OR "revision rate"[tiab] OR reoperation[tiab] OR "range of motion"[tiab] OR "range of movement"[tiab] OR ROM[tiab] OR ASES[tiab] OR Constant[tiab] OR VAS[tiab] OR "patient reported outcomes"[tiab] OR PROMs[tiab] OR complication*[tiab] OR failure[tiab]) |
