# Supplementary material for: Primary reverse total shoulder arthroplasty in patients aged ≤65 years: a systematic review and meta-analysis
Source: JSES Rev Rep Tech. 2026 Mar 19;6(3):100722. doi: 10.1016/j.xrrt.2026.100722 (PMC13092040; doi:10.1016/j.xrrt.2026.100722)
Supplement: Supplementary Table 2 [file mmc2.docx]

| **Supplementary Table 2.** | | | |
| --- | --- | --- | --- |
| Excluded studies with reasons for exclusion | | | |
| No | Title | Year | Reason |
| 1 | Stemless anatomic and reverse shoulder arthroplasty in patients under 55 years of age with primary glenohumeral osteoarthritis: an analysis of the Australian Orthopedic Association National Joint Replacement Registry at 5 years | 2025 | Not all patients have 2 years FU, overlapping cohort with No3,4 |
| 2 | Complication rates after shoulder arthroplasty in patients aged 45 years and younger | 2025 | Not extractable data |
| 3 | Survivorship of shoulder arthroplasty in young patients with osteoarthritis: an analysis of the Australian Orthopaedic Association National Joint Replacement Registry | 2023 | Not all patients have 2 years FU, overlapping cohort with No1,4 |
| 4 | Shoulder joint arthroplasty in young patients: Analysis of 8742 patients from the Australian Orthopaedic Association National Joint Replacement Registry | 2023 | Not all patients have 2 years FU, overlapping cohort with No1,3 |
| 5 | Incidence of primary anatomic and reverse total shoulder arthroplasty in patients less than 50 years of age and high early revision risk | 2023 | Has revision cases. |
| 6 | Outcomes of reverse shoulder arthroplasty in patients under 55 years old: Results from the New Zealand joint registry | 2023 | Not all patients have 2 years FU, overlapping cohort with No7 |
| 7 | The lifetime revision risk of primary anatomic and reverse total shoulder arthroplasty | 2023 | Not all patients have 2 years FU, overlapping cohort with No6 |
| 8 | Clinical outcomes after reverse shoulder arthroplasty in patients 60 years old and younger; medium-term results | 2023 | Has revision cases |
| 9 | Surgical Complications After Reverse Total Shoulder Arthroplasty and Total Shoulder Arthroplasty in the United States | 2021 | Not all patients have 2 years FU |
| 10 | Osteoarthritis of the shoulder in under-50 year-olds: A multicenter retrospective study of 273 shoulders by the French Society for Shoulder and Elbow (SOFEC) | 2021 | Has revision cases. |
| 11 | Clinically relevant results of reverse total shoulder arthroplasty for patients younger than 65 years compared to the older patients | 2021 | Not all patients have 2 years FU |
| 12 | Clinical results of bony increased-offset reverse shoulder arthroplasty (BIO-RSA) associated with an onlay 145° curved stem in patients with cuff tear arthropathy: a comparative study | 2020 | Not extractable data |
| 13 | Do younger patients have better results after reverse total shoulder arthroplasty? | 2018 | Has revision cases. |
| 14 | Early Outcomes Following Metal-on-Metal Reverse Total Shoulder Arthroplasty in Patients Younger Than 50 Years | 2016 | Has revision cases. Not all patients have 2 years FU |
| 15 | Younger patients report similar activity levels to older patients after reverse total shoulder arthroplasty | 2016 | Not all patients have 2 years FU |
| 16 | Clinical outcomes of reverse total shoulder arthroplasty in patients aged younger than 60 years | 2014 | Has revision cases. |
| 17 | Early follow-up of reverse total shoulder arthroplasty in patients sixty years of age or younger | 2013 | Has revision cases. |
| 18 | Reverse total shoulder arthroplasty for massive irreparable rotator cuff tears in patients younger than 65 years old: results after five to fifteen years | 2013 | Overlapping cohort with study by Ernstbrunner et al. (included in our analysis) |
| 19 | Massive rotator cuff tears in patients younger than 65 years. What treatment options are available? | 2009 | Overlapping cohort with study by Berhouet et al. (included in our analysis) |
| 20 | Infected Shoulder Arthroplasty in Patients Younger than 60 Years: Results of a Multicenter Study | 2023 | Has revision cases. |
| 21 | Predictors of Readmission and Reoperation Following Shoulder Arthroplasty in Patients Under 45 Years of Age | 2023 | Has less than 2 years follow-up |
| 22 | Survivorship of Reverse Shoulder Arthroplasty According to Indication, Age and Gender | 2022 | Has revision cases. |
| 23 | The relationship between age and short-term complications following reverse total shoulder arthroplasty for proximal humerus fractures | 2021 | Has less than 2 years follow-up |
| 24 | Salvage reverse total shoulder arthroplasty for failed operative treatment of proximal humeral fractures in patients younger than 60 years: long-term results | 2020 | Has revision cases. |
| 25 | Shoulder arthroplasty in patients 59 years of age and younger | 2013 | Not extractable data |
