# Supplementary material for: Primary reverse total shoulder arthroplasty in patients aged ≤65 years: a systematic review and meta-analysis
Source: JSES Rev Rep Tech. 2026 Mar 19;6(3):100722. doi: 10.1016/j.xrrt.2026.100722 (PMC13092040; doi:10.1016/j.xrrt.2026.100722)
Supplement: Supplementary Table 3 [file mmc3.docx]

| **Supplementary Table 3. Risk of bias assessment using MINORS criteria** | | | | | | | | | | | | | |
| --- | --- | --- | --- | --- | --- | --- | --- | --- | --- | --- | --- | --- | --- |
| **Criteria** | Panel, 2025 | Barry, 2025 | Berhouet, 2024 | Deliso, 2023 | Neel, 2022 | Shah, 2021 | Monir, 2020 | Brewley, 2020 | Matthews, 2019 | Ernstbrunner, 2017 | Samuelsen, 2017 | Otto, 2017 | Black, 2014 |
| A clearly stated aim | 2 | 1 | 2 | 2 | 2 | 2 | 1 | 2 | 2 | 2 | 2 | 2 | 2 |
| Inclusion of consecutive patients | 1 | 0 | 1 | 2 | 1 | 1 | 2 | 2 | 1 | 2 | 1 | 1 | 1 |
| Prospective collection of data | 0 | 0 | 0 | 0 | 2 | 2 | 0 | 2 | 0 | 1 | 0 | 2 | 0 |
| Endpoint appropriate to the aim of the study | 2 | 2 | 2 | 1 | 2 | 2 | 2 | 2 | 2 | 2 | 2 | 2 | 2 |
| Unbiased assessment of the study endpoint | 1 | 1 | 1 | 1 | 1 | 1 | 1 | 1 | 1 | 2 | 1 | 2 | 1 |
| Follow-up period appropriate to the aim of the study | 1 | 2 | 2 | 1 | 2 | 1 | 2 | 2 | 2 | 2 | 2 | 2 | 2 |
| Loss to follow-up less than 5% | 2 | 2 | 2 | 2 | 2 | 2 | 2 | 0 | 2 | 1 | 1 | 2 | 1 |
| Prospective calculation of the study size | 0 | 0 | 0 | 0 | 0 | 0 | 0 | 0 | 0 | 0 | 0 | 0 | 0 |
| Additional criteria in the case of comparative study | | | | | | | | | | | | | |
| An adequate control group | 2 | 2 |  | 1 | 2 | 2 |  | 2 | 2 |  |  |  |  |
| Contemporary groups | 2 | 2 |  | 2 | 2 | 2 |  | 2 | 1 |  |  |  |  |
| Baseline equivalence of groups | 1 | 1 |  | 2 | 1 | 1 |  | 1 | 2 |  |  |  |  |
| Adequate statistical analyses | 1 | 2 |  | 2 | 2 | 2 |  | 2 | 2 |  |  |  |  |
| Maximum possible score | 24 | 24 | 16 | 24 | 24 | 24 | 16 | 24 | 24 | 16 | 16 | 16 | 16 |
| Total MINORS score | 15 | 15 | 10 | 16 | 19 | 18 | 10 | 18 | 17 | 12 | 9 | 13 | 9 |
