# Supplementary material for: Primary reverse total shoulder arthroplasty in patients aged ≤65 years: a systematic review and meta-analysis
Source: JSES Rev Rep Tech. 2026 Mar 19;6(3):100722. doi: 10.1016/j.xrrt.2026.100722 (PMC13092040; doi:10.1016/j.xrrt.2026.100722)
Supplement: Supplementary Table 4 [file mmc4.docx]

| **Supplementary Table 4. Comparative outcomes of primary rTSA in older adults** | | | | | | | | | | | | | | | | |
| --- | --- | --- | --- | --- | --- | --- | --- | --- | --- | --- | --- | --- | --- | --- | --- | --- |
| **ID** | **Study** | **N** | **Mean Age** | **Male/female** | **FU** | **Complications** | **Revision** | **5-year ISR** | **10-year ISR** | **Post-operative** | | | | | | |
|  |  |  |  |  |  |  |  |  |  | **ROM** | **ASES** | **VAS** | **Constant** | **SST** | **UCLA** | **SPADI** |
| 1 | Panel | 30792 | 73 | 10882/ 19910 |  |  | 352 |  |  |  |  |  |  |  |  |  |
| 2 | Barry | 170 | 71.2 | 64/106 | 4.5 | 6.4% | 6 | 96.7% | 94.9% | FE:138±26, ER:39±12, IR:L5 | 75.9±22.3 | 1.7±2.3 |  | 7.7±3.2 |  |  |
| 3 | Deliso | 29 | 74.4 | 6/23 | 3.2 |  | 4 |  |  | FE:112.9±34.2, ER:43.3±17.2, IR:42.1±18.2, ABD:109.1±31.9 |  |  |  |  |  |  |
| 4 | Neel * | 1763 | 71 | 635/ 1128 | 3.9 | 3.34% | 31 |  |  | FE:140±27, ER:38±18, IR:4.5±1.7, ABD:119±31 | 82±20 | 1.3±2.1 | 69±14 | 9.9±2.7 | 30±5.3 | 23±26 |
| 5 | Shah | 76 |  |  |  |  |  |  |  |  | 79±20 |  |  |  |  |  |
| 6 | Brewley ** | 651 |  |  | 4.16 |  | 13 |  |  | FE:141±45.10, ER:51±45.10, IR:4±6.44, ABD:130±48.5 | 72±19.33 |  |  |  |  |  |
| 7 | Matthews *** | 43 | 76.3 | 12/26 | 4.1 | 6.97% | 1 |  |  | FE:121±18.6, ER:31±15.15, IR:L2, ABD:115±26.26 | 79.3±18 |  | 74.5±13.6 | 9.3±2.6 | 29.1±2.1 | 31.4±22.9 |
| N: Number of patients, FU: Follow-up (mean) in years, ISR: Implant survival rate, FE: Forward elevation, ER: External rotation, IR: Internal rotation, ABD: Abduction  *: Internal rotation was measured using a point system, **: Internal rotation was measured using a point system and SD was calculated from mean and 95% CI using the method described in: Higgins JPT, Thomas J, Chandler J, Cumpston M, Li T, Page MJ, Welch VA (editors). Cochrane Handbook for Systematic Reviews of Interventions version 6.5 (updated August 2024). Cochrane, 2024. Available from [www.cochrane.org/handbook](http://www.cochrane.org/handbook)., ***:Internal rotation was measured using a point system and SD was calculated using MD, p-value and t-statistic, as described in: Higgins JPT, Thomas J, Chandler J, Cumpston M, Li T, Page MJ, Welch VA (editors). Cochrane Handbook for Systematic Reviews of Interventions version 6.5 (updated August 2024). Cochrane, 2024. Available from [www.cochrane.org/handbook](http://www.cochrane.org/handbook). | | | | | | | | | | | | | | | | |
