# Supplementary figures and images for: Primary reverse total shoulder arthroplasty in patients aged ≤65 years: a systematic review and meta-analysis
Source: JSES Rev Rep Tech. 2026 Mar 19;6(3):100722. doi: 10.1016/j.xrrt.2026.100722 (PMC13092040; doi:10.1016/j.xrrt.2026.100722)

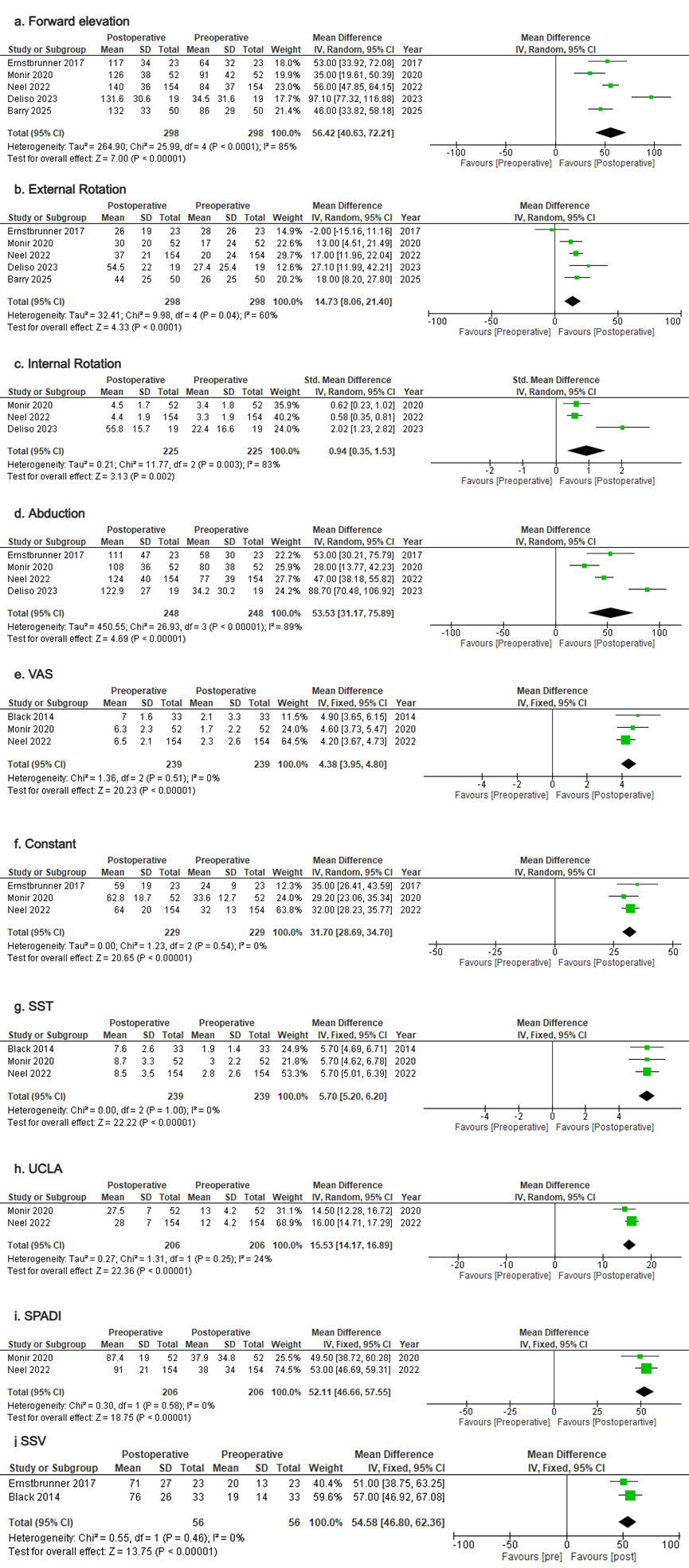


Supplementary figure 7: Sensitivity analysis after removal of studies with imputed data

Supplement: Supplementary Figure 2 [file mmc6.docx]

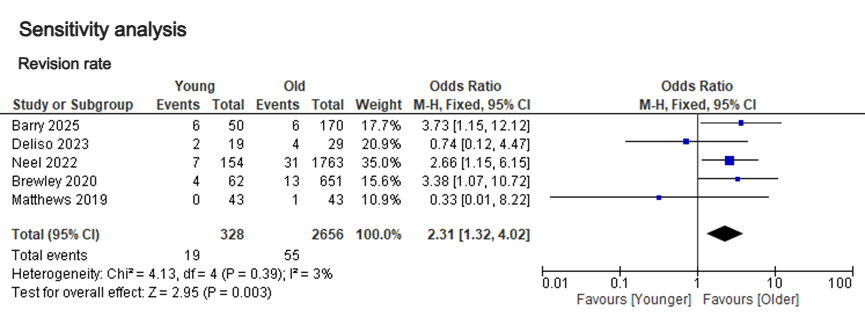

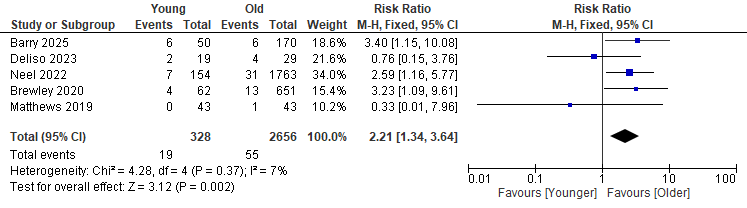


Supplementary figure 6: Sensitivity analysis for revision rate.

Supplement: Supplementary Figure 7 [file mmc11.docx]
