# Supplementary material for: Primary reverse total shoulder arthroplasty in patients aged ≤65 years: a systematic review and meta-analysis
Source: JSES Rev Rep Tech. 2026 Mar 19;6(3):100722. doi: 10.1016/j.xrrt.2026.100722 (PMC13092040; doi:10.1016/j.xrrt.2026.100722)
Supplement: Supplementary Figure 3 [file mmc7.docx]

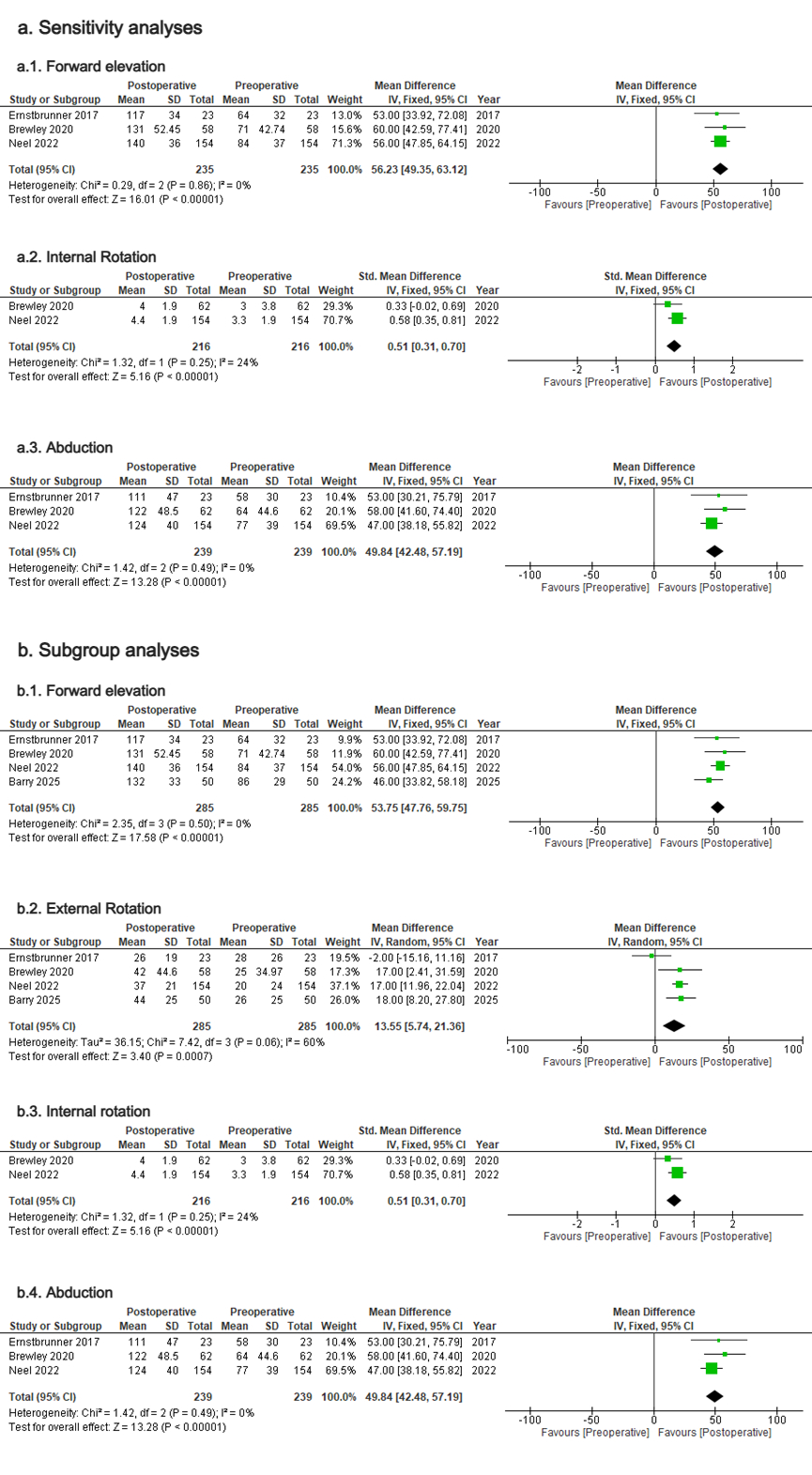


Supplementary figure 2: (a) Sensitivity analyses evaluating postoperative improvements in (a.1) forward elevation, (a.2) internal rotation, and (a.3) abduction after exclusion of studies including fracture indications. (b) Subgroup analyses restricted to studies reporting outcomes for patients <60 years old, in (b.1) forward elevation, (b.2) external rotation, (b.3) internal rotation, and (b.4) abduction.
