# Supplementary material for: Primary reverse total shoulder arthroplasty in patients aged ≤65 years: a systematic review and meta-analysis
Source: JSES Rev Rep Tech. 2026 Mar 19;6(3):100722. doi: 10.1016/j.xrrt.2026.100722 (PMC13092040; doi:10.1016/j.xrrt.2026.100722)
Supplement: Supplementary Figure 4 [file mmc8.docx]

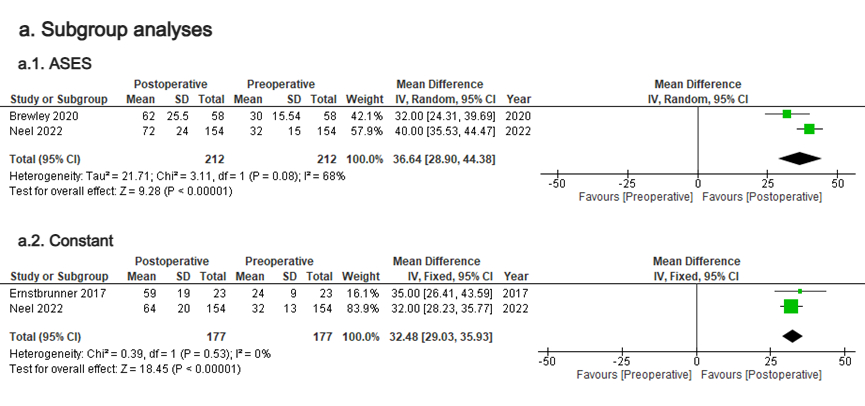


Supplementary figure 3: Subgroup analyses restricted to studies reporting outcomes for patients <60 years old. (a.1) ASES score, (a.2) Constant score.
