# Supplementary material for: Primary reverse total shoulder arthroplasty in patients aged ≤65 years: a systematic review and meta-analysis
Source: JSES Rev Rep Tech. 2026 Mar 19;6(3):100722. doi: 10.1016/j.xrrt.2026.100722 (PMC13092040; doi:10.1016/j.xrrt.2026.100722)
Supplement: Supplementary Figure 5 [file mmc9.docx]

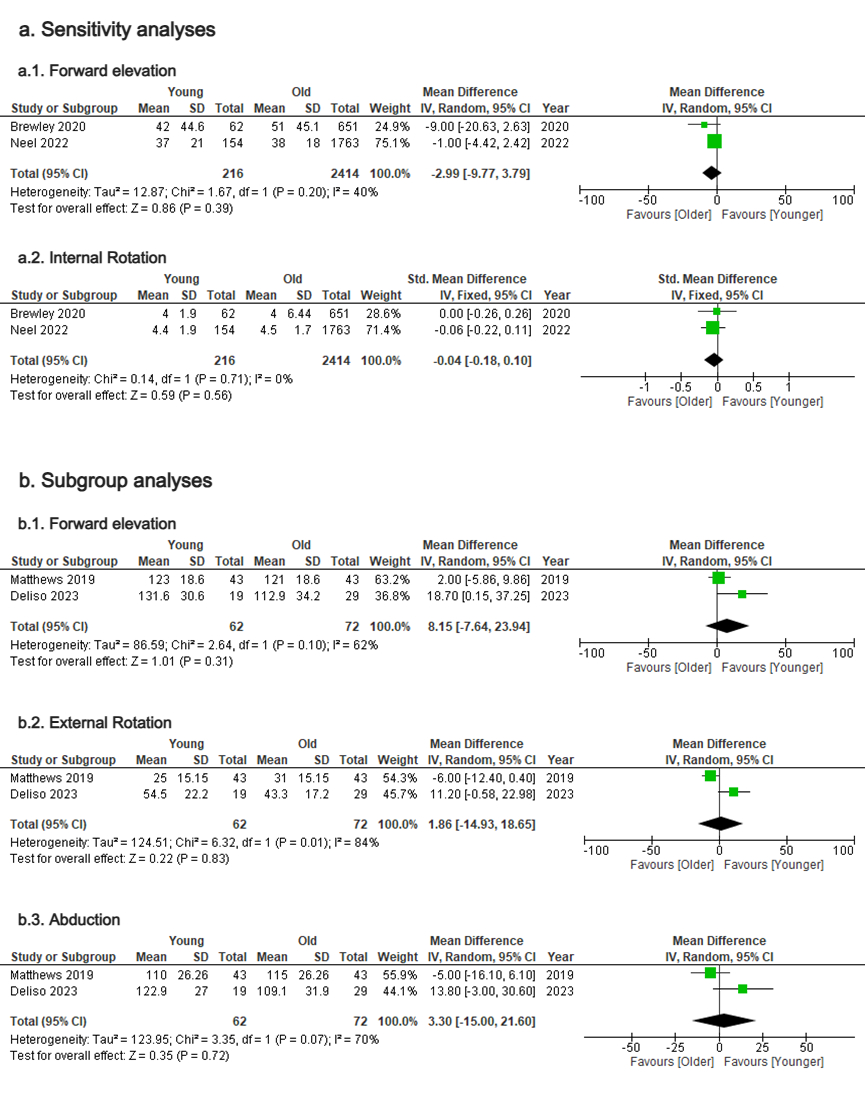


Supplementary figure 4: (a) Sensitivity analyses comparing postoperative ROM outcomes between younger and older adults after exclusion of studies involving fracture indications. (a.1) forward elevation, (a.2) internal rotation. (b) Subgroup analyses based on studies directly comparing postoperative ROM between patients younger than 65 years old and older. (b.1) forward elevation, (b.2) external rotation, (b.3) abduction.
