# Supplementary material for: Primary reverse total shoulder arthroplasty in patients aged ≤65 years: a systematic review and meta-analysis
Source: JSES Rev Rep Tech. 2026 Mar 19;6(3):100722. doi: 10.1016/j.xrrt.2026.100722 (PMC13092040; doi:10.1016/j.xrrt.2026.100722)
Supplement: Supplementary Figure 6 [file mmc10.docx]

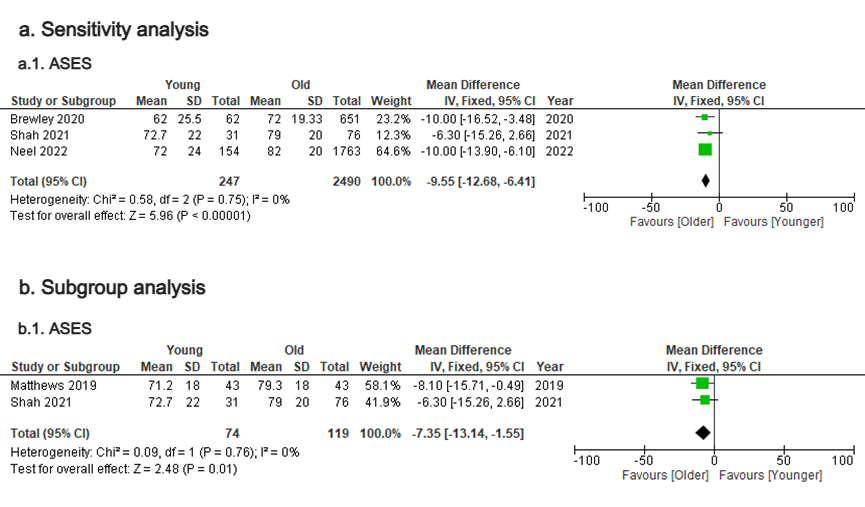


Supplementary figure 5: (a) Sensitivity analysis of postoperative ASES scores comparing younger and older adults, after exclusion of studies involving fracture indications. (a.1) ASES score. (b) Subgroup analysis based on studies directly comparing postoperative PROMs between patients younger than 65 years old and older. (b.1) ASES score.
